# Rush Medical College

**Published:** 1859-09

**Authors:** 


RUSH MEDICAL COLLEGE.—PROF. ALLEN.—If the balance of the new appointments in Rush Medical College are as judicious as that of Prof. Allen to the Chair of Theory and Practice, the School will offer attractions of a very high order; and large classes will be likely to convene annually under the droppings of an institution which adopts the celebrated Rush as its patron Saint. Prof. Allen was identified with the organization of the Medical Department of the University of Michigan, and for four years continued his labors in that institution. As a scientific lecturer, he is, in our judgment, unsurpassed; at least, it has never been our fortune to listen to his superior. His lectures are always strong, clear, and convincing. His style is terse and axiomic. Conceiving in his own mind a clear and definite idea of the subject under consideration, separating truth from error, and reducing facts to general philosophy, he never fails to present truth with a clear and bold outline, and in a highly assimilative form. His acquisition is fortunate for the Rush Medical College; and while we regret that Dr. Allen leaves Michigan, we can but commend the sagacity which secures his services, and express our sincerest wishes for his personal welfare.—G., *Peninsular and Independent Medical Journal.*

